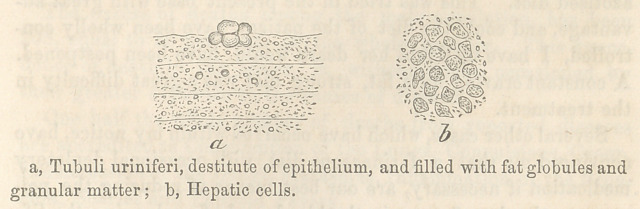# Microscopic Examination of a Case of Morbus Brightii, in Connection with Melanotic Cancer

**Published:** 1854-02

**Authors:** Joseph H. Wythes


*Microscopic Examination of a case of Morbus Brightii, in connection with Melanotic Cancer.* By JOSEPH H. WYTHES, M. D.

In the beginning of April, 1853, Miss Mary Leib, aged 47, put herself under my care. She had been afflicted in one way or other since she was 14 years of age, and had been the most of that time under medical treatment. She was of medium height and stoutness; walked with a slight stoop in her shoulders; had a peculiarly anxious expression of countenance, though of cheerful and patient temper; had always been temperate; was in easy circumstances, and had not ceased to menstruate.

She complained of palpitation of the heart, shortness of breath, and of swelling in her ankles and feet. On examination, I found the heart's action full, strong, and heaving, diffused throughout the chest, its rythm altered, and its sound muffled, as if oppressed. Lungs dull on percussion, and respiration impeded. I judged the case to be one of hydrothorax, with hypertrophy and dilatation of the left ventricle of the heart. The lower extremities were swollen from dropsical effusion.

I gave her a pretty active purge of jalap and cream of tartar, and directed a pill, containing digitalis and squill, each half a grain, to be taken three times a day. After the dropsical symptoms had subsided, I ordered tinct. digitalis, 10 drops, three times a day, with a view to moderate the heart's action. This treatment appeared to answer my expectations and was discontinued.

In a few days afterwards, I found my patient in an attack of epileptic convulsions, which soon wore off. As she had complained, previously, of pain in the back, I was led to suspect granular degeneration of the kidneys, and on testing the urine with heat and nitric acid, I found it very greatly albuminous.

Attacks of dropsical effusion and epilepsy succeeded each other for several months, yielding, apparently, to treatment, which was varied according to circumstances. Sometimes, when indicated by a full, strong pulse, venesection afforded great relief to the oppressed lungs. In a similar case, which I attended in company with another physician, so great was the relief afforded by venesection, that the patient rode, the next day, upon horseback, upwards of 40 miles, with no immediate ill consequence, though he died within a month.

On Oct. 31st, I desired a consultation with Dr. Carpenter, of Pottsville. The patient was laboring under great oppression, from hydrothorax, with comatose symptoms; and ordinary purgatives, &c., failed to operate. We agreed to use Elaterium 1-16th gr., Calomel 1 gr., Jalap 2 grs., in a pill, to be given two or three times a day. This having no effect save the production of nausea, I altered it to Elaterium 1-5th gr., Bi-Tart. potass 3 grs., Pulv. Zingiber 2 grs. She took this three times a day, for several days, with but slight (if any) effect on the bowels, although the effusion in the chest and limbs evidently decreased, and the patient seemed better. She so far recovered as to be able to go about the house. On the 20th of November she was suddenly attacked with dyspnœa and coma; in which condition she died the next morning.

At the request of the family, I made a post-mortem examination on the 22d, being assisted by Drs. G. W. Brown and J. I. Wright.

The outside of the body presented nothing peculiar, except a fluctuation, on pressure, at the base of the chest. The blood in the veins was in a somewhat fluid state, for the bandage having been removed from the arm of the corpse, (where she had been bled on the 20th,) a considerable quantity of blood had escaped.

On opening the chest, a large quantity of serous fluid was observed within the pleura. We took from thence 20 teacupsful—about equal to 120 f.℥. There were also very extensive fibrous adhesions of the pleura on the left side.

The lungs were congested with venous blood, and contained small, melanotic tumors, about the size and consistence of a grain of wheat. The pericardium contained about 2 f.℥ of bloody serum.

The heart was much enlarged—considerable dilatation of the left ventricle with hypertrophy of its walls. The muscular parietes of the right ventricle softened, so that the finger could be pushed through. The semi-lunar valves slightly thickened at the margin. The aorta much dilated, with patulous congestion of the internal membrane.

The kidneys were distinctly granular in the cortical portion, and their surface contained a number of serous cysts, varying in size from that of the head of a pin to that of a filbert.

The intestines externally and the bladder appeared quite healthy.

On the posterior surface of the uterus was a tumor, about the size of a filbert, which, on cutting, proved quite hard and fibrous, with a coarse granular appearance, having a cyst in the middle, full of black fluid. Similar melanotic cysts, with thinner parietes were observed in the ovaries, particularly the right ovary.

A microscopic examination of a thin section of the tumor from the uterus, showed a number of fusiform cells passing into fibres, which still retained their neuclei; parent-cells, containing each a number of smaller cells, most of which showed both neuclei and neucleoli ; fat globules ; numerous smaller fat granules ; ovoid, oblong, polyhedral and caudate cells, generally neucleated ; and melanotic pigment in dark granules, floating in an amorphous liquid. The melanotic matter from the ovaries, had few fusiform cells, but cells containing black pigment were more numerous.

The accompanying drawing (fig.1) shows the form of these products magnified 250 diameters.

a, fusiform or fibrous cells ; b, parent-cells ; c, cells with neuclei, but without neucleoli ; d, fat globules and granules ; e, smaller cells of various shapes ; f, pigment cells and granules.

The vessels of the liver and kidney were injected, the former with size, the latter with the ethereal injection, as suggested by Dr. Goddard of Philadelphia, that having been found by me, usually, more penetrating than any other kind of minute injection.

The renal artery was filled with fine vermillion. The veins were injected with blue. On a subsequent examination, it was found that the investing tunic was strongly adherent to the surface of the kidney, which presented a mottled or marbled appearance, from the number of yellowish and apparently fatty granules which covered it.

A few, only, of the Malpighian bodies received the arterial injection, and these were disorganized, looking rather like small tufts than a convolution of vessels. The veins of the surface presented, in many parts, the peculiar stellated arrangement which has been described as characteristic of the latter stages of Bright's disease.

A thin section of the uninjected kidney showed the uriniferous tubes, deprived of epithelium, and covered with fat globules and granular matter.

The hepatic cells of the liver were covered with granular matter, and exhibited but few neuclei or oil-globules. The accompanying illustration (fig. 2) shows the appearance of both liver and kidney under the microscope.

*a* *b*

a, Tubuli uriniferi, destitute of epithelium, and filled with fat globules and granular matter; b, Hepatic cells.

If, as suggested by Mr. Bowman, the Malpighian bodies in the kidney, serve for the elimination of the watery part of the urine, it is easy to see how their obliteration causes the accumulation of watery fluid in other parts of the body. May not the melanotic cysts be also accounted for by the increased exhalation, without corresponding absorption, arising from this cause?

The excess of urea in the blood, as shown by the albuminous urine, sufficiently accounts for the epileptic and comatose symp toms, while the enlargement and thickening of the left ventricle of the heart, may have been caused by the increased demand upon its functions. Not only may the degeneration of the kidneys have contributed to this latter effect, by causing an engorgement of the vessels, but the abnormal stimulus of the urea in the blood, would tend to accelerate the heart's action. It appears, therefore, a primary indication to lessen, if possible, the amount of urea in the blood.

The case before us is eminently instructive, as confirmatory of the views of Dr. Johnson, Mr. Toynbee, and other eminent pathologists, respecting lesions of the kidney, and their influence on the general health. Whether these lesions can be detected in their early stages, and anything done towards a radical cure, is a question worthy of much regard; the more so as disorders of this kind are far from being uncommon.

The experiments of Dr. Lehman of Leipsic, upon himself, have clearly proved that the amount of urea eliminated by the kidneys in health, is, to a great extent, dependent on the character of the diet; the urine abounding in urea during the use of animal food, while it is very considerably diminished under a non-azotised diet. It would therefore be proper to confine a patient suffering under disorganization of the kidneys to a purely farinaceous or non-azotised diet. This was tried in the present case with great advantage, and could the diet of the patient have been wholly controlled, I have no doubt her death would have been postponed. A constant craving for a fat, strong diet was a great difficulty in the treatment.

Several other cases, which have occurred within my notice, have convinced me that a farinaceous diet, with occasional depletory medication if necessary, are our best means of reducing the poisonous stimulus of urea in the blood, and of prolonging the life of the patient.

## Figures and Tables

**Figure f1:**
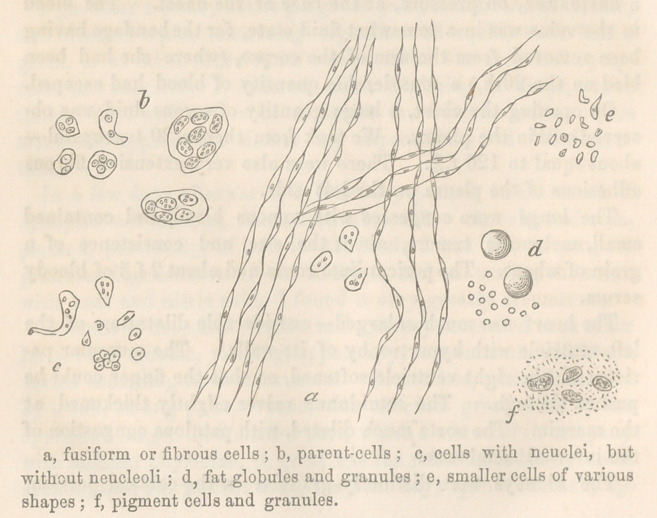


**Figure f2:**